# Heredity and Cancer

**Published:** 1915-07

**Authors:** 

**Affiliations:** San Antonio


### The Department of Eugenics, Mental and Social Hygiene

CONDUCTED BY
DR. MALONE DUGGAN
813-814 Gibbs Building, San Antonio, Texas
All communications for this department should be sent to DR. DUGGAN

### Heredity and Cancer.

That cancer has long been regarded as influenced by heredity is shown in our almost universal custom of including it in our case histories and in the requirement of life insurance examinations. To be a causative agent, it must be proven that the cancer cell is a heritable unit. This has never been done in man, and the assumption, that it is so, has rested, heretofore, purely on empirical grounds.

But, very recently, investigations on mice have apparently proven the assumption to be true, at least so far as mice are concerned. Miss Maud Slye, of the Sprague Memorial Institute, by very extensive investigations, concludes that cancer is a mode of growth which is profoundly influenced by the ancestry of the mice, and that the power to resist the cancer is a Mendelian unit. This acts as any other heritable unit, and can be bred into or bred out of at will. The author produced strains of mice in which cancer was the usual cause of death, and, again, other strains in which cancer was unusual. She further transmitted the non-cancerous strains into the cancerous strains, and the reverse. The hybrids of this propagation proved to act in accordance with Mendelian principles.

From this demonstration the author deduces the fact that the liability to cancer is the thing that is transmitted. This means that when the liability is present, with appropriate conditions, cancer will result. In other words, heredity modifies the character or degree of the effect produced by a common injury. By analogy, then, we may conclude that what has been found to be true in relation to mice is also true as regards mankind.

